# Feelings, Stress, and Adaptation Strategies of Nurses against COVID-19 in Guayaquil

**DOI:** 10.17533/udea.iee.v38n3e07

**Published:** 2020-11-11

**Authors:** Joicy Anabel Franco Coffré, Patricia de los Ángeles Leví Aguirre

**Affiliations:** 1 Nurse, Master’s. Assistant Full-Time Professor. Email: joicy.francoc@ug.edu.ec joicy.francoc@ug.edu.ec; 2 Nurse, Master’s of Science. Principal Professor (Retired). Email: songuest@yahoo.com songuest@yahoo.com; 3 Nursing Career, Universidad de Guayaquil, Ecuador. Universidad de Guayaquil Universidad de Guayaquil Ecuador

**Keywords:** Stress, psychological, nurses, coronavirus infections, risk factors, pandemics, cross-sectional studies, Ecuador, estrés psicológico, enfermeras y enfermeros, infecciones por coronavirus, factores de riesgo, pandemias, estudios transversales, Ecuador, estresse psicológico, enfermeiras e enfermeiros, infecções por coronavirus, fatores de risco, pandemias, estudos transversais, Ecuador

## Abstract

**Objective.:**

To explore the feelings, stress factors, and adaptation strategies of nurses during the COVID-19 pandemic in Guayaquil, Ecuador.

**Methods.:**

A cross-sectional, descriptive quantitative study, conducted through the application of a 52-item questionnaire with four sections (feelings, perceived stress, stress-reducing factors, and adaptation strategies). The study population was 227 nursing professionals from “Hospital General del Guasmo Sur” of the Ministry of Public Health, who worked during the peak of the pandemic from March to May 2020. The sample comprised 155 nurses who voluntarily accepted to participate. The study received 127 complete questionnaires for analysis.

**Results.:**

The data showed the priority of humanist feelings and professional duty for these nurses, mostly young (59% under 35 years of age and with the professional exercise of three and fewer years), against the fear of contagion and the stress of strenuous work. They also revealed the great importance for them of the institutional support, recognition to the staff, and strict organization of safe care, like strategies for coping with this difficult experience.

**Conclusion.:**

The COVID-19 pandemic represented for nurses from Guayaquil a great professional and emotional challenge. Health services and society could consider these findings to avoid burning out nurses and their professional desertion.

## Resumen

**Objetivo.:**

Explorar los sentimientos, factores de estrés y estrategias de adaptación de los enfermeros durante la pandemia del COVID-19 en Guayaquil, Ecuador.

**Métodos.:**

*.* Estudio cuantitativo, descriptivo, de corte transversal, realizado mediante la aplicación de un cuestionario de 52 ítems con cuatro secciones (sentimientos, estrés percibido, factores reductores de estrés y estrategias de adaptación). La población del estudio fueron 227 profesionales de enfermería del “Hospital General del Guasmo Sur” del Ministerio de Salud Pública, que laboraron durante el pico de la pandemia en los meses de marzo a mayo de 2020. La muestra fueron 155 enfermeros que aceptaron voluntariamente participar. Se recibieron 127 cuestionarios completos para el análisis.

**Resultados.:**

Los datos mostraron la prioridad de los sentimientos humanistas y del deber profesional para estos enfermeros, en su mayoría jóvenes (59% menores de 35 años y con ejercicio profesional de tres y menos años), frente al temor al contagio y al estrés de un trabajo extenuante. Revelaron también la gran importancia que tiene para ellos el soporte institucional, el reconocimiento al personal y la estricta organización de una prestación segura, como estrategias para el afrontamiento de esta difícil experiencia.

**Conclusión.:**

La pandemia del COVID-19 representó para los enfermeros de Guayaquil un gran desafío tanto profesional como emocional. Los servicios de salud y la sociedad podrían considerar estos hallazgos para evitar el desgaste de los enfermeros y su deserción profesional.

## Resumo

**Objetivo.:**

Explore os sentimentos, fatores de estresse e estratégias de adaptação de enfermeiras durante a pandemia COVID-19 em Guayaquil, Equador.

**Métodos.:**

Estudo quantitativo, descritivo, transversal, realizado por meio da aplicação de um questionário de 52 itens com quatro seções (sentimentos, estresse percebido, fatores redutores do estresse e estratégias de adaptação). A população do estudo foi de 227 profissionais de enfermagem do “Hospital Geral de Guasmo Sur” do Ministério da Saúde Pública, que trabalharam durante o pico da pandemia nos meses de março a maio de 2020. A amostra foi de 155 enfermeiros que aceitaram participar voluntariamente. Foram recebidos para análise 127 questionários completos.

**Resultados.:**

Os dados evidenciaram a prioridade do sentimento humanístico e do dever profissional para esses enfermeiros, em sua maioria jovens (59% menores de 35 anos e com prática profissional de até três anos), frente ao medo do contágio e ao estresse do trabalho extenuante. Também revelaram a grande importância do apoio institucional, do reconhecimento da equipe e da estrita organização de uma prestação segura, como estratégias para o enfrentamento dessa difícil experiência.

**Conclusão.:**

A pandemia COVID-19 representou um grande desafio profissional e emocional para as enfermeiras de Guayaquil. Os serviços de saúde e a sociedade poderiam considerar essas descobertas para evitar a exaustão do enfermeiro e sua deserção profissional.

## Introduction

In December 2019, an infection outbreak occurred due to Severe Acute Respiratory Syndrome - Coronavirus 2 (SARS -COV-2) in the city of Wuhan, province of Hubei, China.(1) The global advance of COVID-19 was vertiginous; by January it had spread to Thailand and Spain; in February it was in Italy and Egypt and in March, it reached Latin America, where one of the first countries affected was Ecuador (29 February 2020).(2) Due to antecedents of high contagion, lethality in European countries, and vulnerability of the Ecuadorian health system, local authorities initiated follow up of contacts, detection, and control of cases. On 17 March, the Ecuadorian government took restrictive measures and declared the state of emergency, confinement of the citizenship, and border closings to contain the advance of the virus(3) and the Ministry of Health selected hospitals for referral of patients with COVID-19.

Structural changes were established immediately in health services and reforms were made in the staff’s workload, principally due to reasons of logistics and staffing (schedules from 8 to 12 hours daily changed to 24-hour shifts). The vast expansion of COVID-19 in a few days in Guayaquil did not permit timely supply of personal protection equipment, or diagnostic tests, which had very slow processing due to insufficient capacity of daily testing.(4) By late March, the installed capacity of hospitals and clinics in the city was exceeded and even the capacity of funeral services collapsed. By 15 May 2020, 30,502 confirmed cases were reported and 2,338 deaths.(5) At the time, Ecuador had the highest rate of cases in South America, 13.15 per 100-thousand inhabitants, which exceeded the global average of 9.63 and pointed to growing lethality.(6)

The institutional response for nurses upon this complex crisis was based only on their personal protection and biosecurity measures. There was no regard to the unprecedented consequences to the mental health of the health staff. Pandemics not only cause disease and death, they also cause prolonged grief, fear, despair, hopelessness, sleep disorders, and post-traumatic stress that lead to significant social, occupational, and interpersonal dysfunction.(7) The COVID-19 pandemic represented a great threat to the lives and health of the staff. In Ecuador, according to data from professional colleges, there were 700 physicians infected, 80 dead; 147 nurses infected and 8 dead, which caused a significant impact on their emotional responses and on their mental health.(5,7)

Lazarus and Folkman(8) defined stress “as the interrelations between the person and the context, produced when the *person* evaluates that which happens as something exceeding the resources they have available and endangers their personal wellbeing”. Additionally, these authors proposed a new concept, “coping with stress”, according to which each person has a particular way of coping that can be determined by the health and physical energy, existential beliefs, motivations, capacity for problem solving, control of the environment and the very person, and social support.

Some studies exist on stress, coping and mental health of nurses at risk of COVID-19, one of them in Anhui, China evidenced that nurses showed anxiety, fear, anger, and sadness.(9) In Huazhong, it was found that 35 members of the front-line nursing staff (85.37%), with patients in severe phase of pneumonia due to COVID-19, experienced somatizations, obsessive-compulsive symptoms, interpersonal sensitivity, depression, anxiety and fear.(10) An Iranian study identified that nurses in the first line of care to COVID-19 patients had a higher job-stress level than those working in other services.(11) Studies have also identified stress reducing factors and adaptation strategies in the health staff in the pandemic: availability of personal protection equipment, rigorous protocols to control infections, recognition of their efforts by the hospital administration and government, and decrease of new cases reported, which provide them psychological benefit.(12)

The nursing staff in the front-line of care faces large and varied demands due to contact with very ill patients with a contagious virus and of poorly known trajectory; the vulnerability not only in them, but also the possibility of bringing the virus into their homes; observing the deaths of patients alone; and varied information projected in communication and social media, that do not encourage them at all.(12,13) Therein the importance of establishing in nurses a baseline of factors related to mental health to implement actions that improve the Nursing exercise against new outbreaks.

## Methods

This study had a cross-sectional, descriptive approach. It was conducted in “Hospital General Guasmo Sur” with a capacity of 474 beds, which was designed as the first hospital in the city of Guayaquil to care for patients during the peak of the COVID-19 pandemic, from March to May 2020. The universe of nursing professionals working in the hospital was of 267 of which 40 were excluded due to having criteria of vulnerability for the disease, resulting in a population of 227, distributed in the services of Emergency (79), ICU (35), Hospitalization, and others (113). Given the pandemic circumstances, a non-probability convenience sampling was carried out, recruiting the professionals who voluntarily accepted to participate in the study. Of the 227 nurses who worked during the pandemic, 155 answered the questionnaire, but only 127 fully completed it, with these being the ones finally analyzed. It is important to consider that all the research subjects lived the same experience, given that they exclusively cared for patients with COVID-19 of slight, moderate, and severe symptomatology, guaranteeing non-variability of the result due to the participants.

Data collection was performed by applying an on-line questionnaire on the *Survey Monkey* ^®^ platform. Given that at the time of executing the survey there was mobility restriction and social distancing, an invitation was delivered to the professional nursing staff via e-mail to participate in the research. The invitation explained the objective and characteristics of the study, guaranteeing anonymity and confidentiality and included a link with the questionnaire form. The informed consent was taken as implicit if the participants connected to the web site and completed the questionnaire. Instructions were attached for the on-line survey, which indicated how the questions were distributed, the meaning of each item and its corresponding scale.

The instrument used for this study was a questionnaire derived and adapted from the “MERS-CoV staff questionnaire” by Khalid *et al.,*(13) selected because its variables have much affinity with those of the impact of COVID-19 on the health staff during the pandemic in Guayaquil. After obtaining authorization from the author, it was translated into Spanish by one of the authors of this article for greater reliability in the connotations and affirmations. Additionally, the content was analyzed with six local experts (four nurses and two psychologists who work with Nursing), who individually evaluated the questionnaire and then participated in two group meetings on the Zoom platform, to detect ambiguities and remove that which was not in context or which was not pertinent for the objective of this research, resulting in 52 items among reactions and behaviors.

The questionnaire had four sections with the following response options: (i) *emotions of nurses during the COVID-19 pandemic* with 12 items and its scale: 0 = not at all; 1 = somewhat; 2 = moderate; 3 = very much); (ii) *stress-causing factors* with 16 items and its scale: 0 = no stress; 1 = light stress; 2 = moderate stress; 3 = too much stress; (iii*) factors that help to diminish the stress of nurses* with 11 items and its scale: 0 = not effective; 1 = slightly effective; 2 = moderately effective; 3 = very effective; and (iv) *coping strategies the staff could have used* with 13 items and their scale: 0 = never used; 1 = sometimes used; 2 = frequently used; 3 = always used. The study processed 127 complete questionnaires, which were analyzed with descriptive statistics. Kuder Richardson-Formula 20 and Cronbach’s alpha internal consistency coefficients were also obtained. The SPSS statistical program version 25 was used. The questionnaire was subjected to a pilot test with a group of 10 nursing professionals with practice similar to the study subjects, modifications were not needed.

## Results

In this study, it was interesting that the sample was mostly comprised of young nurses, seven in every ten were under 35 years of age, and 58% had a time of professional exercise of three years and less; 85.8% were women,74% had no graduate formation, 71% practiced a religion, and 89% lived with the family (Table 1).

**Table 1:** General characteristics of the study sample (*n*=127)

Characteristic	Category	Value	%
Age (years)	20 to 24	15	11.81
	25 to 29	47	37.01
	30 to 34	28	22.05
	35 to 39	1	11.02
	40 to 44	11	8.66
	45 to 49	3	2.36
	50 and more	9	7.09
Sex	Female	109	85.83
	Male	18	14.17
Service working in	Emergency	42	33.07
	Hospitalization	37	29.13
	Intensive Care Unit	36	28.35
	Other	12	9.45
Level of education	Degree in Nursing	94	74.02
	Specialist	2	1.57
	Masters	6	4.72
	Others: diploma courses	25	19.69
Years of clinical experience	0 to 3	74	58.27
	4 to 8	32	25.20
	9 to 12	5	3.94
	13 and more	16	12.60
Practices some religion	Yes	90	70.87
Family situation	Lives with the children	12	9.45
	Lives with the spouse	17	13.39
	Lives with the spouse and children	50	39.37
	Lives alone	14	11.02
	Lives with others	34	26.77

The feelings manifested most by the nursing staff during the pandemic were the professional duty of the nurses, 91% (score = 2.33); fear of caring for patients, 91%, (score = 1.80); and the appreciation they would give to institutional recognition, 89%, (score = 2.30). In lower proportion, they felt dissatisfaction for having to work extended hours, 34%, (score = 0.54) or tried to diminish their contact with patients. Very few wanted to call in sick, 7%, (score = 0.11) or felt the urge to quit their job (Table 2).

**Table 2:** Emotions of nurses during the pandemic (*n* = 127, maximum score = 3)

Number	Emotions	Responded yes (%)	Average score (±SD)
1	Did you feel you had to do your job when caring for patients with COVID-19 as a professional duty?	91	2.33 (0.96)
2	Did you feel frightened when caring for patients with COVID-19?	91	1.80 (0.89)
3	Were you upset because your workload increased with patients with COVID-19?	54	0.91 (0.97)
4	Were you dissatisfied working an extended schedule (increased number of hours in your work shift)?	34	0.54 (0.83)
5	Did you try to diminish your contact with COVID-19 patients (for example, going less to the patient’s unit)?	42	0.69 (0.90)
6	Have you felt that relatives, friends, or neighbors avoid contact with you?	85	1.70 (1.06)
7	Have you felt the urge to quit your job due to the pandemic?	29	0.50 (0.89)
8	Would you appreciate a special recognition from the hospital administration due to your work with COVID-19 patients?	89	2.30 (1.02)
9	Would you appreciate an economic compensation for caring for COVID-19 patients?	80	1.81 (1.19)
10	If it were optional, would you have elected working in a unit where you would not be exposed to COVID-19?	42	0.68 (0.93)
11	Have you thought of calling in sick to avoid going to work?	7	0.11 (0.44)
12	Did you feel exhausted or fatigued due to your work with COVID-19 patients?	85	1.33 (0.87)

Score key: 0 = Not at all; 1 = Slightly; 2 = Moderately; 3 = Very much

Among the factors that cause stress, we may highlight due to its frequency and intensity, the possibility of transmission to relatives, 99%, (score = 2.27), as well as factors related to the workplace, such as getting infected by handling patients, 94%, (score = 1.90); and lack of personal protection equipment, 91%, (score = 2.02). An external factor, lack of treatment and vaccines available for this virus, 91%, (score = 2.01), proved stressful. Results above 88% were obtained in various factors: accessing news through television or social media about COVID-19; observing anxious and frightened colleagues, and having possible symptoms of the disease (Table 3).

**Table 3:** Factors that caused stress among nursing professionals (*n* = 127, maximum score = 3)

Number	Factors	Responded yes (%)	Average score (±SD)
1	Were you stressed regarding the probability of transmitting COVID-19 to your family or friends due to your work?	99	2.27 (0.76)
2	Were you stressed when having to care for your own colleagues, acquaintances, or relatives sick with COVID-19?	80	1.43 (0.97)
3	Were you stressed by having to deal with the deaths of patients with COVID-19?	83	1.80 (1.10)
4	Were you stressed by the probability of contracting the COVID-19 infection by handling patients in the hospital?	94	1.90 (0.90)
5	Were you stressed by the lack of adequate protection measures (sufficient equipment and adequate environments) against COVID-19?	91	2.02 (0.98)
6	Were you stressed by the fear of making minimal mistakes or failures in concentration during work, which would expose you or others to COVID-19 infection?	86	1.52 (0.93)
7	Were you stressed by a conflict between your professional duty and your own safety?	75	1.32 (1.02)
8	Were you stressed by the fact of having to use protection equipment daily (discomfort)?	83	1.54 (1.01)
9	Were you stressed by news of new COVID-19 cases reported on TV/newspapers/social media?	88	1.76 (1.01)
10	Were you stressed by the probability of enduring rejection or attacks from the community?	78	1.37 (0.99)
11	Were you stressed when seeing your unit’s colleagues anxious or frightened?	88	1.48 (0.88)
12	Were you stressed when seeing colleagues who showed symptoms similar to those of COVID-19?	89	1.61 (0.94)
13	Were you stressed by the fact of having respiratory symptoms and fearing it was COVID-19?	89	1.88 (1.01)
14	Were you stressed by having to take the test to detect the COVID-19 infection after being exposed?	84	1.62 (1.02)
15	Were you stressed by the lack of a treatment and/or vaccine for COVID-19?	91	2.01 (0.97)
16	Were you stressed by not knowing when there would be more COVID-19 outbreaks?	88	1.72 (0.98)

Score key: 0 = No stress; 1 = Slightly stressed; 2 = Moderately stressed; 3 = Very stressed

Among the factors that aided in diminishing stress, it was found that all the nurses benefit when all the colleagues in the unit where they work show a positive attitude, 100%, (score = 2.56), there is teamwork within the area, 100%, (score = 2.76), knowing that COVID-19 cases improve, 100%, (score = 2.69), and no one is sick among their family and circle of friends, 100%, (score = 2.74). A lower percentage (84%) reported that it helps not having an extended schedule at work (more hours in the work shift) (Table 4).

**Table 4:** Factors that help to reduce stress in nursing professionals (*n* = 127, maximum score = 3)

Number	Factors	Responded yes (%)	Average score (±SD)
1	It helps to diminish stress when your unit’s colleagues display a positive attitude.	100	2.56 (0.67)
2	It helps to diminish stress when no COVID-19 cases occur in the staff after applying rigorous protection measures.	98	2.39 (0.77)
3	It helps to diminish stress by observing improvement in the health condition of patients with COVID-19.	100	2.69 (0.60)
4	It helps to diminish stress with the hospital providing individual protection equipment.	97	2.54 (0.82)
5	It helps to diminish stress by knowing that their relatives or friends outside the hospital have not gotten sick with COVID-19.	100	2.74 (0.57)
6	It helps to diminish stress by listening to the news reporting a decrease of new COVID-19 cases.	97	2.32 (0.82)
7	It helps to diminish stress by thinking of the probability additional compensation for their work during the COVID-19 pandemic.	95	2.16 (0.92)
8	It helps to diminish stress when all first-line of care health professionals work as a team.	100	2.76 (0.55)
9	It helps to diminish stress by knowing there is trust in the support to the hospital staff in case they get sick or die from COVID-19.	94	2.34 (0.91)
10	It helps to diminish stress when not having an extended Schedule at work (more hours than the regular).	84	1.74 (1.05)
11	It helps to diminish stress when getting free food in the hospital or in the unit.	91	1.98 (1.00)

Score key: 0 = Not effective; 1 = Slightly effective; 2 = Moderately effective; 3 = Very effective

The coping strategies adopted by all the nurses during the pandemic were, in the first place, those related with the safety of their practice: following strictly personal protection measures, 100%, (score = 2.92); maintaining separate clothing for the street and for work, 100%, (score = 2.81); and acquiring greater knowledge about the disease, 100%, (score = 2.63). Together with these strategies, there was another prevention measure disseminated globally, like avoiding going to public places, 100%, (score = 2.69). High percentages were obtained in the communication strategies with their relatives and friends; positive thoughts and attitudes, as well as caring for their nutrition, frequency of engaging in physical exercise and recreation activities. The lowest percentage and intensity value were obtained in the expression of their feelings, 61%, (score = 0.94) (Table 5).

**Table 5:** Coping strategies used by nursing professionals to reduce stress (*n* = 127, maximum score = 3)

Number	Strategies	Responded yes (%)	Average score (±SD)
1	Follow strict personal protection measures (*e.g*., hand washing, using a face mask, taking the temperature, using disposable gowns).	100	2.92 (0.27)
2	Maintain separate clothing for street and work to minimize transmission.	100	2.81 (0.48)
3	Engage in healthy activities, like a balanced diet, exercise, rest, recreation, etc.	98	2.21 (0.83)
4	Actively acquire greater knowledge about COVID-19, its symptoms, prevention, transmission mechanisms, and management, etc.	100	2.63 (0.53)
5	Avoid going to public places to minimize exposure to COVID-19	100	2.69 (0.54)
6	Practice relaxation activities (*e.g*., meditation, yoga, prayer, dance, others).	90	1.74 (0.96)
7	Talk to relatives and friends to relieve stress and get support.	98	2.38 (0.74)
8	Renovate thoughts and get motivated to face the COVID-19 pandemic with a positive attitude.	99	2.46 (0.70)
9	Obtain help from doctors and professionals from the hospital to reduce stress and remain calm.	93	1.87 (0.93)
10	Seek distraction and try to stay busy at home with activities that distance you from information about COVID-19.	93	2.00 (0.93)
11	Avoid working extended schedules to reduce exposure to COVID-19.	66	1.28 (1.10)
12	Avoid news in the media about COVID-19 and related deaths.	89	1.79 (0.97)
13	Express your emotions by crying, yelling, etc.	61	0.94 (0.95)

Score key: 0 = Never used; 1 = Sometimes used; 2 = Frequently used; 3 = Always used

Lastly, the Cronbach’s alpha coefficient, used for scales of continuous items, showed that the internal consistency of the section of *Stress-causing factors* was high, while it was moderate in the other three. The Kuder-Richardson test coincided with that reported for the Cronbach’s alpha in the section of *Stress-causing factors* as high consistency and of *Emotions of nurses during the pandemic* as moderate, while in the other sections the internal consistency was interpreted as low (Table 6).

**Table 6:** Internal consistency coefficient of the sections of the instrument used

Section	Kuder-Richardson 20	Cronbach’s alpha
Emotions of nurses during the pandemic	0.77	0.72
Stress-causing factors	0.87	0.93
Factors that aid in reducing stress in nursing professionals	0.47	0.77
Coping strategies the staff could have used	0.55	0.73

## Discussion

The COVID-19 pandemic placed health professionals throughout the world in a situation without precedent. The impact in Guayaquil, city where the pandemic began in Ecuador, was devastating.(2) Current scientific literature reports that work under pressure, administration and optimization of scarce resources in services, fear of death, and placing one’s basic needs aside due to overload of responsibilities, additionally, could affect the physical state and mental health of nurses.(9,10,12-14) Perhaps the more robust health systems have the power to better mitigate impacts, versus weaker health systems, as could be the case in Ecuador.(3) To better explain it, the Ecuadorian National Health System has a more curative than preventive orientation. Regarding the need for better detection, follow up, and control of cases in the first level of care, the conditions did not exist for this to take place in the city of Guayaquil and patients had to attend hospital institutions of greater complexity. As a result, greater saturation was observed of users, work overload, and contagion of health workers.

This study, by being the first to explore the feelings, stress factors, and coping strategies upon COVID-19 used by Ecuadorian nurses, can be a contribution to develop care programs for the staff and have better strategic planning in future outbreaks. The professionals in this study turned out to be, mostly, young recently graduated and with poor work experience who, like many nurses globally, felt that caring for patients with COVID-19 represented a professional duty,(12-14) in spite of their fear of infection, and the extenuating fatigue represented by working with these patients. Fear in 91% of them was higher compared to that young nurses from Taiwan (43%) felt during the possible pandemic of an unknown virus (avian flu), already, warning of the risk of progression from this fear to panic, and then to phobia.(15) A high proportion of the sample (89%) considered institutional recognition important (promotions, job stability, mentions of their performance) and economic compensation for their work. These data are similar to those found among 534 health professionals who were in the front line in Wuhan during the COVID-19 outbreak,(12) and in another study in China.(16)

The factors that caused the greatest stress to the nurses surveyed were the possibility of being infected with COVID-19 and infecting their relatives and friends (99%); likewise, this occurred among nurses in Taiwan with higher intensity (2.35).(17) A critical factor, lack of sufficient personal protection equipment (91%), further complicated by long work shifts that in the “Hospital del Guasmo” were of 24 hours, caused exhaustion and subordination of basic needs, with high physical and psychological demands, and scant resources.(17-19) This situation was, also manifested in Chinese(16) and European hospitals (20) during the peak of the pandemic. Negative news of the advance of COVID-19, as well as the nervousness and anguish of their coworkers, were very important in this group, unlike the study by Khalid on the MERS epidemic outbreak, probably due to its lesser social impact.(13) The perception of greater risk due to the lack of a vaccine and treatment caused stress in 91% of the respondents.

Four of the factors that helped to reduce stress during the peak of the pandemic were similar in various studies, positive attitude from coworkers and team work;(18,20) observing the clinical improvement of hospitalized patients; and having their relatives and friends free from COVID-19.(12,18) Diminished work shift, as well as receiving compensation or free food were of lesser interest. It is noted in this study that the staff has high solidarity and good disposition to provide their professional services despite the overwhelming situation they encountered.

The coping strategies most used by Ecuadorian nurses included: following strict personal protection measures at work and at home; social distancing (avoiding going out to the streets and public places); and acquiring actively greater knowledge about COVID-19 and its management; similar to those of other professionals in epidemics.(12,17,18) Speaking with relatives and friends relieved stress, as well as renovating thoughts and motivating themselves positively served them as support.(18,19) A large group of nurses did not reduce their schedules to protect themselves (34%) nor expressed their emotions openly by crying and yelling (39%), possibly due to the big moral demands of the crisis.(14) The profound ethical and vocational commitment nursing has, is definitely one of the characteristics these professionals have, and the obligation of offering their knowledge and skills during this threatening moment, was fair and pertinent. It was the moment to demonstrate who is who.

According to the results of this research, it is concluded that the impact on the mental health of nurses could be severe. It must be remembered that they not only experience the stress caused by their work on the front line, but that they are also parents, spouses, sons and daughters, and citizens who are experiencing fear and vulnerability regarding this unprecedented situation for the current world. Fortunately, the social and family components influenced favorably the ways of coping of these professionals during the pandemic; besides the positive work environment, resulting in support for their emotional state. Institutional recognition, more than economic compensation, would have greater importance in their perceptions.

Nurses need government and those in charge of formulating public policy, nursing leaders, as well as professional groups to participate actively in their support, both during and after the pandemic^.^ (14,16,19-21) The Union of Nurses from Guayas is monitoring the outfitting of sufficient quality biosecurity supplies for nursing professionals, as well as ensuring possible compensation to the families of nurses who have lost their lives during the pandemic.

This study had limitations with the sample coming from a single hospital and due to the self-selection. Another factor could be the memory bias in the responses upon the fact that the survey took place in late May, after almost two months in which Guayaquil suffered the peak of the pandemic, and “fear” of the virus could have been lost. More studies are recommended from these results, in another group of professionals or in cities of Ecuador that are still in the pandemic cycle.

## References

[1] Zu ZY, Jiang MD, Xu PP, Chen W, Ni QQ, Lu GM, Zhang LJ (2020). Coronavirus Disease 2019 (COVID-19): A Perspective from China. Radiology.

[2] Guerrero S (2020). Coronavirus en Ecuador: una opinión desde la Academia. La Granja.

[3] Hallo A, Rojas A, Hallo C (2020). Perspective from Ecuador, the Second Country with More Confirmed Cases of Coronavirus Disease 2019 in South America: A Review. Cureus.

[4] Bajaña I (2020). Incidencias del COVID-19 en Ecuador. Question/Cuestión.

[5] Riesgos Ecuador (2020). Coronavirus Ecuador - información verificada de la llegada del COVID-19 al país.

[6] Inca GP, Inca AC (2020). Evolución de la enfermedad por coronavirus (COVID-19) en Ecuador. La Ciencia al Servicio de la Salud y la Nutrición.

[7] Levin J (2019). Mental Health Care for Survivors and Healthcare Workers in the Aftermath of an Outbreak. Psychiatry of Pandemics.

[8] Lazarus RS, Folkman S (1986). Estrés y procesos cognitivos.

[9] Huang L, Ming Xu F, Rong Liu H (2020). Emotional responses and coping strategies of nurses and nursing college students during COVID-19 outbreak. medRxiv.

[10] Xu M, Zhang Y, Union Hospital, Tongji Medical College, Huazhong University of Science and Technology (2020). Psychological status survey of first clinical first-line support nurses fighting against pneumonia caused by a 2019 novel coronavirus infection. 护理研究.

[11] Hoseinabadi T, Kakhki S, Teimori G, Nayyer S (2020). Burnout and its influencing factors between front-line nurses and nurses from other wards during the outbreak of Coronavirus Disease (COVID-19) in Iran. Invest. Educ. Enferm..

[12] Cai H, Tu B, Ma J, Chen L, Fu L, Jiang Y, Zhuang Q (2020). Psychological Impact and Coping Strategies of Frontline Medical Staff in Hunan Between January and March 2020 During the Outbreak of Coronavirus Disease 2019 (COVID-19) in Hubei, China. Med. Sci. Monit..

[13] Khalid I, Khalid TJ, Qabajah MR, Barnard AG, Qushmaq IA (2016). Healthcare Workers Emotions, Perceived Stressors and Coping Strategies During a MERS-CoV Outbreak. Clin. Med. Res..

[14] Greenberg N, Docherty M, Gnanapragasam S, Wessely S (2020). Managing mental health challenges faced by healthcare workers during COVID-19 pandemic. BMJ.

[15] Tzeng HM, Yin CY (2006). Nurses' fears and professional obligations concerning possible human-to-human avian flu. Nurs. Ethics..

[16] Liu Y, Aungsuroch Y (2019). Work stress, perceived social support, self‐efficacy and burnout among Chinese registered nurses. J. Nurs. Manag..

[17] Feng MC, Wu HC, Lin HT, Lei L, Chao CL (2020). Exploring the Stress, Psychological Distress, and Stress-relief Strategies of Taiwan Nursing Staffs Facing the Global Outbreak of COVID-19. Hu Li Za Zhi.

[18] Koh D, Lim MK, Chia SE, Ko SM, Qian F (2005). Risk perception and impact of Severe Acute Respiratory Syndrome (SARS) on work and personal lives of healthcare workers in Singapore: what can we learn?. Med. Care..

[19] Mo Y, Deng L, Zhang L, Lang Q, Liao C, Wang N (2020). Work stress among Chinese nurses to support Wuhan in fighting against COVID-19 epidemic. J. Nurs. Manag..

[20] Maben J, Bridges J (2020). COVID‐19: Supporting nurses’ psychological and mental health. J. Clin. Nurs..

[21] Fernandez PR, Lord H, Halcomb PE, Moxham PL, Middleton DR, Alananzeh DI (2020). Implications for COVID-19: a systematic review of nurses’ experiences of working in acute care hospital settings during a respiratory pandemic. Int. J. Nurs. Stud..

